# High Temperature Long Period Grating Thermo-Mechanically Written

**DOI:** 10.3390/s90705649

**Published:** 2009-07-15

**Authors:** Jose Miguel Lazaro, Antonio Quintela, Jose Miguel Lopez-Higuera

**Affiliations:** Photonics Engineering Group, University of Cantabria, Avda. de los Castros S/N, 39005 Santander, Spain; E-Mails: antonio.quintela@unican.es (A.Q.); miguel.lopezhiguera@unican.es (J.M.L.-H.)

**Keywords:** long period gratings, mechanical technique, fabrication, permanent LPG, high temperature sensor

## Abstract

An optical fiber transducer able to work in high temperature environments is experimentally demonstrated in the laboratory. It is based on a permanent long period grating (LPG) written using a new technique based on a thermo-mechanical approach. Device precision was experimentally checked by means of repetitive thermal cycles between 25 and 950 °C. In addition device stability was assured by maintaining the temperature in steady state at 800 °C during 23 hours.

## 1. Introduction

Optical fiber sensors are used in a wide variety of application fields (chemistry, civil engineering, biomedicine, etc.) because of their advantages compared with conventional ones (small size, interference immunity, possibility of long distance interrogation, etc.) [1]. A lot of techniques are used in these sensors (intensity, interferometry, spectrometry, polarimetry, etc), the majority of them based on optical fiber grating structures. In addition, these devices are able to obtain wavelength multiplexing and hence quasi-distributed sensor networks can be built [1].

High temperature sensing is of great interest for many industrial applications and it can be done using fiber grating based transducers. However, when typical grating structures are placed in high temperature environments, they suffer a dramatic degradation on their optical behaviours (as it happens with the ones written using UV photosensitivity technique) and, hence, their usefulness for this purpose is drastically reduced.

Gratings with a period longer than the interrogation wavelength, the so-called long period gratings (LPGs), can be used in transmission configuration to measure temperature with high resolution. Some techniques have been developed in order to make LPGs able to work at high temperatures as using a CO_2_ laser [2] or a Ti:sapphire 800 nm laser [3]. In general, the reported approaches to obtain high temperature stability are based on custom fibers like fibers with a special doping [4–9].

Mechanical LPGs are made by applying pressure with a periodical grooved plate located over a fiber region [10]. In these cases the LPG disappears when the pressure is removed and, due to the plate expansion, this LPG type is unable to work at high temperatures.

Hiscocks *et al.* [11] made a permanent grating by means of heating a mechanical LPG in a microstructured polymer optical fiber. The written LPG can not be used at high temperatures due to the lower melting point of the polymer. Also, the fabrication is easier than in a standard fiber due to the different melting point of silica and polymer.

In this paper, an inexpensive thermo-mechanical technique is proposed to fabricate permanent LPG structures on standard telecommunication fibers that are able to work at high temperatures. With this technique the use of intense lasers, dopants or other fiber pre-processing to increase the photosensitive are avoided. In the following, theory, fabrication technique, experimental works, results and discussion are presented. Finally some conclusions are outlined.

## 2. Theory

The LPG periodicity (typically between 100 μm and 1 mm) produces a coupling between the fundamental propagation mode and the forward propagating cladding modes. This coupling follows the phase matching condition given, for each mode, in [12]:

(1) $$\lambda_{\mathrm{res}}^{i}=\left(n_{\mathrm{eff}}\;\left(\lambda \right)-n_{\mathrm{cladd}}^{i}\;\left(\lambda \right)\right)\Lambda$$

where $\lambda_{\mathrm{res}}^{i}$ is the coupled wavelength, *n_eff_* is the effective index of the fundamental core mode, $n_{\mathrm{cladd}}^{i}$ is the effective index of the i^th^-order cladding mode and Λ is the period of the core refractive index modulation or grating period. The cladding modes present high propagation attenuation and, as a consequence, the spectral response of a LPG shows several attenuation peaks centred at the coupled wavelengths between fundamental core mode and a cladding mode [12].

**Figure 1.**
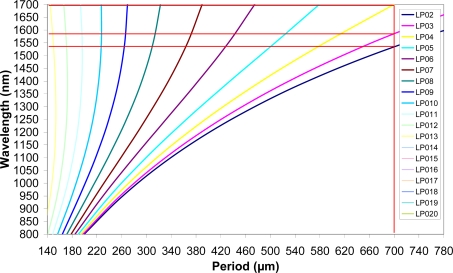
Coupling wavelength as a function of period for different cladding modes in a LPG on a standard telecommunication fiber. (It has been obtained from the simulation of the core and cladding effective indexes).

Due to the dependence of the effective index on the wavelength, the interrelation between coupled wavelength and period is not linear, as can be observed in Figure 1. For a given LPG period, Λ, the resonant wavelengths of each mode, $\lambda_{\mathrm{res}}^{i}$, can be obtained. Thus for the period used in our device (700 μm) three peaks are expected to be obtained at 1,530, 1,590 and 1,700 nm, respectively.

## 3. Experimental Set-Up

**Figure 2.**
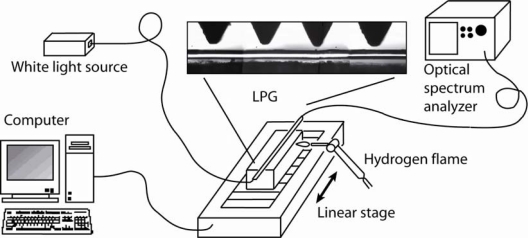
Illustration of the experimental set-up used to write thermo-mechanically LPGs.

A periodically grooved plate is located and pushed with a fixed force over a region of the standard fiber. Next, it is heated with a very localized hydrogen flame and, hence, the groove shapes are permanently written on the fiber. As it can be observed in Figure 2, the hydrogen flame (∼2,000 °C) sweeps the fiber-grooves region using a linear stage controlled by a computer. In this way, the LPG profile can be even apodized. In order to follow the writing process in real-time, the LPG optical behaviours are monitorized using a broadband light source (Agilent 83437A) and an optical spectrum analyzer (HP86142A). The source consists of 4 appropriate LEDs covering the spectral range from 1,200 to 1,700 nm.

## 4. Results and Discussion

**Figure 3.**
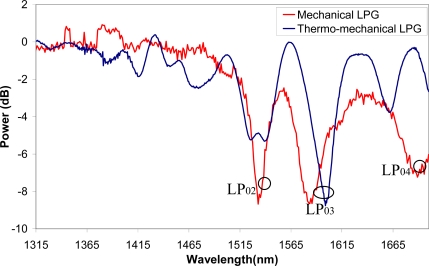
Temporal LPG spectral response induced by mechanical pressure (red) and permanent LPG spectral response with the thermo-mechanical technique (blue).

As shown in Figure 3 (in red) the transmission spectrum response of a mechanically induced LPG (pushing a grooved plate of 700 μm period and 5 cm length over the fiber) is in agreement with the simulations.

Next, in order to permanently write the LPG on the fiber, sweeps were carried out with the hydrogen flame and, in this way, the spectral response shown in Figure 3 (blue colour) is obtained. It can be noticed that, as expected, the spectrum remains unaltered after removing the grooved plate. A small shift on the attenuation peaks is observed with respect to the previous case. Additional small peaks are also obtained but they can be removed with the technique refinement and using its apodization capability.

The device temperature behaviour was measured checking the LPG by means of a furnace (Carbolite MTF 12/38/250). The LPG was placed inside the tube-furnace measuring the temperature with a thermocouple situated near the fiber device.

**Figure 4.**
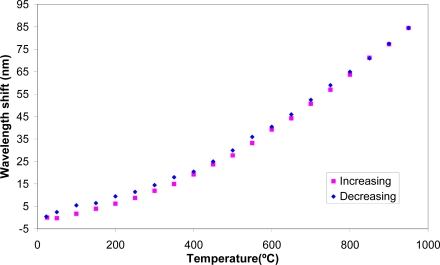
Evolution of the peak wavelength as a function of the temperature. Increasing (pink) and decreasing (blue) the temperature.

Temperature sweeps between 25 to 950 °C and *vice versa* were carried out and results are summarized on Figure 4. An attenuation peak shift of ∼85 nm in the mentioned temperature range was encountered. The peak returns to the original position what means that no appreciable hysteresis effect (with the temperature) is observed in this LPG device.

Finally, the transmission spectrum stability of the LPG working at a high temperature was also characterized. The LPG was subjected to 800 °C during 23 hours and the attenuation peak remained practically unaffected. Therefore, it is possible to conclude that the grating is not removed with high temperature. The LPG characteristics remained after this period.

## 5. Conclusions

A permanent LPG able to work at high temperature environments has been written using a thermo-mechanical technique. A hydrogen flame has been applied to a fiber with a periodical grooved plate over it and, thus, the fiber structure has been modified according to the plate pattern and the flame heat which allow the possibility of apodize the grating shape. A shift of ∼ 85 nm in the attenuation peak wavelength with temperature (25 to 950 °C) has been measured and no hysteresis has been observed. Very good stability of the attenuation peak has been obtained maintaining the LPG to 800 °C during several hours. The results suggest that useful LPGs for fiber devices and sensors able to work at high temperature environments can be fabricated with this technique.
